# Targeting the Endocytic Pathway and Autophagy Process as a Novel Therapeutic Strategy in COVID-19

**DOI:** 10.7150/ijbs.45498

**Published:** 2020-03-15

**Authors:** Naidi Yang, Han-Ming Shen

**Affiliations:** 1 Key Laboratory of Flexible Electronics (KLOFE) & Institute of Advanced Materials (IAM), Nanjing Tech University (NanjingTech), 30 South Puzhu Road, Nanjing, Jiangsu Province 211800, China; 2 Faculty of Health Sciences, University of Macau, Macau SAR, China

**Keywords:** Coronaviruses, endocytic pathway, autophagy, SARS-CoV-2, COVID-19

## Abstract

Coronaviruses (CoVs) are a group of enveloped, single-stranded positive genomic RNA viruses and some of them are known to cause severe respiratory diseases in human, including Severe Acute Respiratory Syndrome (SARS), Middle East Respiratory Syndrome (MERS) and the ongoing coronavirus disease-19 (COVID-19). One key element in viral infection is the process of viral entry into the host cells. In the last two decades, there is increasing understanding on the importance of the endocytic pathway and the autophagy process in viral entry and replication. As a result, the endocytic pathway including endosome and lysosome has become important targets for development of therapeutic strategies in combating diseases caused by CoVs. In this mini-review, we will focus on the importance of the endocytic pathway as well as the autophagy process in viral infection of several pathogenic CoVs inclusive of SARS-CoV, MERS-CoV and the new CoV named as severe acute respiratory syndrome coronavirus 2 (SARS-CoV-2), and discuss the development of therapeutic agents by targeting these processes. Such knowledge will provide important clues for control of the ongoing epidemic of SARS-CoV-2 infection and treatment of COVID-19.

## Brief introduction of the new coronaviruses

Coronaviruses (CoVs) are enveloped viruses with a long single-stranded RNA ranging from 26 to 32 kilobases (kb) in size 1. CoVs belong to the family *Coronaviridae* in the order *Nidovirales,* and have been organised into 3 groups: α-CoVs, β-CoVs, and γ-CoVs 2. Two of the β-CoVs including severe acute respiratory syndrome coronavirus (SARS-CoV) and Middle East respiratory syndrome coronavirus (MERS-CoV) caused severe acute respiratory disease outbreaks in China in 2002-2003 and in the Middle East in 2012, respectively 3.

In December 2019, a novel CoV outbreak, identified and named as severe acute respiratory syndrome coronavirus 2 (SARS-Cov-2) started in Wuhan, Hubei province, China. The SARS-CoV-2 spread very quickly in China and then to the many other countries, causing coronavirus disease-19 (COVID-19). The clinical futures of COVID-19 mainly include fever, cough and pneumonia 4. Up to date, it has already infected more than 90,000 people worldwide and killed more than three thousand patients, mainly in Wuhan, China. SARS-Cov-2 shares a high sequence identity (around 80%) with SARS- CoV and a 96.2% sequence identity with BatCoV RaTG13, a bat CoV 5. Although some initial cases were linked to a local seafood market in Wuhan, its origin, intermediate hosts and how it was transmitted to humans are still largely unknown 4.

In this mini-review, we will mainly focus on β-CoV, which is inclusive of SARS-CoV, MERS-CoV, and the current emerging SARS-CoV-2 to discuss the implication of the endocytic pathway and autophagy process in the infection of these pathogenic CoVs and therapeutic potential of targeting these processes. This review will also include the well-studied mouse hepatitis virus (MHV) since it is often used as a safe mode to study CoV infection.

## Brief introduction of the autophagy and the endocytic pathway

**Figure 1:**
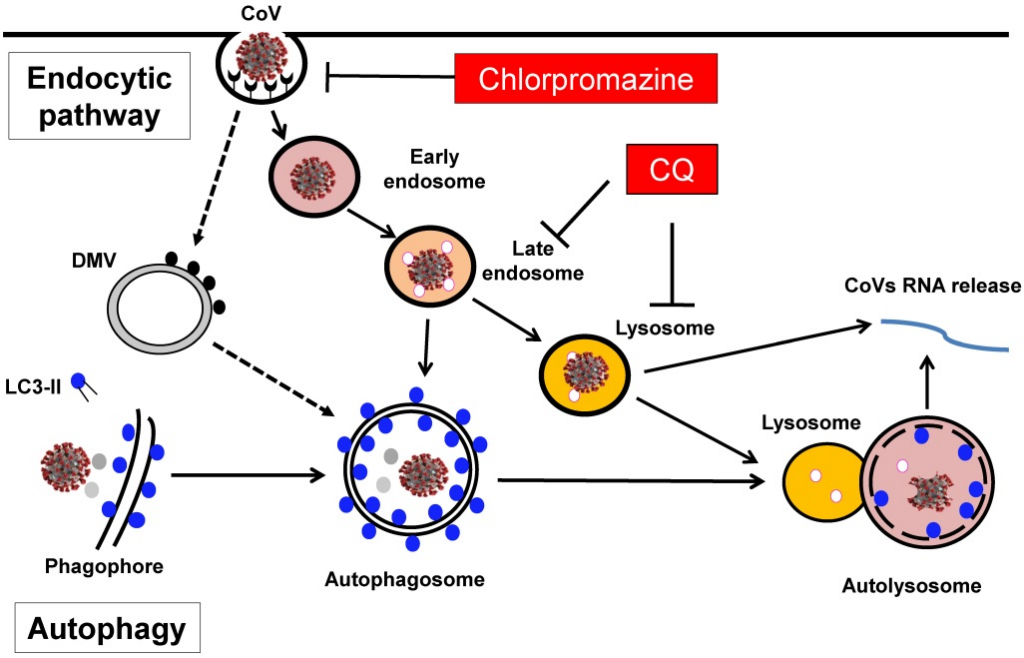
** Involvement of the endocytic pathway and autophagy in the entry and replication of CoVs in host cells.** Entry of CoVs into the host cells is mainly mediated by the endocytic pathway, meanwhile the autophagy has also been implicated in the viral replication in the cells, a process partly related to the formation of DMV in the host cells. As a result, several groups of inhibitors including the lysosomotropic agents such as CQ and inhibitors for clathrin-mediated endocytosis such as chlorpromazine have been proposed to have therapeutic efficacy against CoVs-induced diseases including COVID-19.

Macroautophagy or autophagy refers to an evolutionarily conserved process in which the intracellular components such as protein aggregates and damaged organelles are engulfed into a double-membrane structure called autophagosome, which eventually fuses with lysosome to form autolysosome for degradation 6, 7 (**Figure 1**). The whole autophagy process is controlled by a group of proteins encoded by autophagy-related-genes (*ATGs*) in several consecutive stages 8, 9. First, the *induction or initiation* stage is controlled by the ULK1/Atg1 complex, downstream of the mechanistic target of rapamycin complex 1 (mTORC1). Second, the *nucleation/expansion/elongation* stage is mediated by the ATG14-Beclin1-hVPS34/class III phosphatidylinositol 3-kinases (PI3K) complex, as well as the two ubiquitin-like conjugation systems (ATG5-ATG12 and LC3/ATG8). The third and last stage of autophagy is the *maturation/fusion/degradation* in which autophagosome fuses with lysosome to form autolysosome where the luminal contents are degraded (**Figure 1**). At present, the biological functions of autophagy have been extensively studied. Autophagy plays an important role in various physiological and pathological processes, including cell survival, cell death, aging, immunity and metabolism 10, 11. More importantly, accumulating evidence has highlighted the importance of autophagy in many human diseases, such as cancer, neurodegenerative diseases, metabolic disorders, as well as immunity and infection 12, 13. Among them, the implication of autophagy in viral infection has also been widely investigated and deeply appreciated.

In the course of autophagy, lysosome plays an essential role in the maturation/degradation stage of autophagy, as the contents in the autophagosomes are eventually degraded by lysosomes, via autophagosome-lysosome fusion 14-16. Lysosome, first discovered by the Nobel laureate Christian de Duve in the 1950s, is the most important digestive organelle present in almost all eukaryotic cells and with an array of important biological functions, including endocytosis, exocytosis, macropinocytosis, plasma membrane repair, defense against pathogens, cell death, signal transduction, and autophagy 14, 17. Lysosome is featured by its acidic internal pH which is generated by the action of a V-ATPase, a proton-pumping membrane protein complex 18. Lysosome contains more than 50 acid hydrolases, including proteases, peptidases, phosphatases, nucleases, glycosidases, sulfatases, and lipases designated for all types of macromolecules 19.

On the other hand, lysosome is also a key component of the endocytic pathway, also termed as the endolysosomal network 20 (**Figure 1**). In addition to autophagy as described above, lysosome receives cargos from other processes including endocytosis through the endocytic pathway. Briefly, following endocytosis, internalized cargos first enter the early endosomes (EE) where the cargos are sorted for two destinations: they are either retrieved for recycling to the plasma membrane/the secretory pathway, or are delivered to the late endosome (LE) and then fuse with lysosome for degradation 20, 21. The main biological functions of the endocytic pathway are for retrieval and recycling of internalized cargo proteins, and such functions are known to play critical roles in the pathology of important human diseases, in particularly neurodegenerative diseases and viral infection 22, 23.

## Implication of autophagy in CoVs infection

In the past one and a half decades, the implication of autophagy in CoV infection has attracted substantial attention, probably due to the SARS outbreak in 2002-2003 and the emerging field of autophagy research at the same period. At present, various reports have converged onto two important questions: whether CoV induces autophagy and whether the autophagy machinery or ATG proteins are involved in the infection and replication of CoVs. The first report demonstrating the involvement of autophagy in viral replication was based on MHV 24, in which the authors made several important observations. First, MHV induced the formation of double-membrane vesicles (DMVs), with resemblance to autophagosome, a hallmark of autophagy. Second, the viral replication complexes at DMVs co-localized with the autophagy proteins, LC3 and ATG12. Third and more importantly, MHV replication was impaired in *ATG5* knockout embryonic stem cells. Therefore, the authors concluded that autophagy is implicated in the formation of DMV as well as in the replication of MHV 24. In a follow-up study, the same group also examined the SARS-CoVs and found similar colocalization of the key viral replication proteins with endogenous LC3, a protein marker for autophagosome 25, suggesting a similar function of autophagy in the replication of SARS-CoVs. Cottam *et al* used another CoV (infectious bronchitis virus, IBV) and found that one of the key viral replicase protein nsp6 is capable of inducing autophagy 26. Notably, this nsp6 also presents in MHV and SARS-CoV, and thus it would be of interest to further test the effects of nsp6 in these two CoVs on autophagy.

However, several subsequent studies have challenged the notion that autophagy is implicated in CoV infection. For instance, in Vero cells infected with SARS-CoVs, Snijder *et al* failed to detect colocalization of LC3 or GFP-LC3 with the viral replication- transcription complexes of SARS-CoV examined using immunofluorescence staining 27. Further studies also demonstrated that either ATG5 or ATG7, two of the key autophagy proteins in control of autophagosome biogenesis, is not required for viral replication in cells infected by MHV 28, 29 or by SARS-CoVs 30. In those studies, cells with deletion of either ATG5 or ATG7 failed to impair the viral replication rate. Similarly, virus infection was not inhibited by the knockdown of ATG5 26. Thus, all these observations suggest that the autophagy machinery is not directly implicated in the viral replication process.

Intriguingly, there is evidence suggesting the possible inhibitory effect of CoV on the autophagy process. For instance, a study using SARS-CoV and MERS-CoV in HEK293T, HeLa and MCF-7 cells found that overexpression of membrane-associated papain- like protease PLP2 (PLP2-TM) of SARS-CoV and MERS-CoV led to blockage of autophagosomes- lysosomes fusion and suppression of the autophagic flux 31. Consistently, a more recent report found that MERS-CoV blocks the fusion of autophagosomes and lysosomes and induction of autophagy reduces the replication of MERS-CoV 32. Thus, it appears that there is certain type of interplay between the autophagy machinery and CoVs, and the exact nature of such interaction remains to be further elucidated.

Taken together, as summarized in **Table 1**, it is still controversial whether and how autophagy is implicated in the infection of CoVs. The discrepancies in the literature is probably due to the different viruses used, different cells tested and even the different techniques used in study of autophagy. More work is thus needed to clarify those important issues.

## Involvement of the endocytic pathway in CoVs infection

One of the key determining factors in viral infection is the entry of the virus into the host cells. At present, it is widely believed that CoVs enter the host cells via two routes: (i) the endocytic pathway and (ii) non-endosomal pathway 33, as partly illustrated in **Figure 1**. Among them, the endocytic pathway is considered to be particularly important and has been extensively studied. As discussed earlier, CoVs are enveloped and plus-strand RNA virus and they contain a set of four proteins that encapsidate the viral genomic RNA: the nucleocapsid protein (N), the membrane glycoprotein (M), the envelope protein (E), and the spike glycoprotein (S) 34. Among them, the function of the S protein is mainly involved in the process of viral entry into the host cells via proteolytic cleavage to form two subunits, S1 and S2 35. These two subunits have distinct functions: S1 is responsible for receptor-binding, while S2 is mainly for membrane fusion and both are essential for viral entry via the endocytic pathway and infection into the host cells.

The first report showing the relevance of endosome-lysosome in CoVs was from a morphological study in which two CoVs (IBV and Porcine Epidemic Diarrhea Virus (PEDV)) were found to accumulate in the lysosomes of cells after infection 36, indicating that the possible functional implication of lysosome in CoVs infection. Subsequent studies have firmly established that the endocytic pathway is the key mechanisms controlling the entry of CoVs into the host cells, and thus the endocytic pathway has been widely investigated as the target of anti-viral therapies, as summarized in **Table 2**.

Among all these studies, there are several important points to be highlighted. First, different CoVs including MHV, SARS-CoV and MERS-CoV have been consistently demonstrated to engage the endocytic pathway as the main mechanism for viral entry into a variety types of host cells. Among them, clathrin-dependent endocytosis and cathepsin- mediated S protein cleavage are two critical steps for the viral entry and infection. In fact, this mechanism is also applicable to many other CoVs such as IBV 37, which is out of the scope of this review.

Second, despite the general understanding for the role of the endocytic pathway for viral entry, there are discrepancies of the exact mechanisms among different reports even with the same CoV. For instance, Wang *et al* found that SARS-CoVs engage clathrin- and caveolae-independent endocytic pathway as the key mechanism for viral entry 38, which is inconsistent with an earlier report in which SARS-CoV entry into HepG2 cells is mostly mediated by the clathrin-dependent pathway 39. Part of the reason for such discrepancies is the different cell types used in their studies, as shown in **Table 2**. Therefore, it is possible that the exact nature of the entry is context-dependent, including the type of the virus and the type of the cells.

Third, at present, the entry mechanisms and the implication of the endocytic pathway of the new emerging SARS-CoV-2 have not been reported directly. It is now known that SARS-CoV-2 utilizes the same receptor of SARS-CoV, which is angiotensin converting enzyme II (ACE2) for viral entry into the host cells 5, 40. ACE2 transcripts was originally only found in heart, kidney and testis of human 41. However, it was later found that ACE2 protein expresses abundantly in the epithelia of the human lung and small intestine 42. Since SARS-CoV-2 also binds to the same ACE2 receptor as SARS-CoV 5 and SARS-CoV-2 is also susceptible to the inhibitory effect of chloroquine (CQ), a lysosomotropic agent 43, it is highly possible that this new CoV utilizes the same endocytic pathway for entry into the host cells. Understanding this mechanism is important in the search of effective therapeutic agents in the treatment of COVID-19 caused by this new CoV.

Finally, in almost all the studies listed in **Table 2**, different inhibitors of the endocytic pathway have been used in blocking viral entry and infection. Among them, three groups of inhibitors are believed to be particularly important and clinically relevant. The first group are the lysosomotropic agents which are capable of accumulating inside and neutralizing the endosome-lysosomal acidic pH and thus blocking the protease activity to inhibit the viral entry. In this group, CQ is an ancient anti-malaria drug and clinically available, as shown in **Figure 1**. The second group are direct endosomal-lysosomal protease inhibitors such as E64d. And the third group are inhibitors for the clathrin-mediated endocytosis such as chlorpromazine, which is also clinically available (**Figure 1**). The details of such inhibitors are to be discussed in the section below.

Taken together, establishing the role of endocytic pathway in viral entry is a major breakthrough in the mechanistic understanding of the CoVs infection, which offers great opportunity in development of novel therapeutic strategies for treatment of diseases such as SARS and COVID-19.

## Targeting the endocytic pathway and autophagy as a novel therapeutic strategy against CoVs

### Lysosomotropic agents targeting endosomal/lysosomal pH

CQ, a well-known anti-malarial drug, is probably the most well-studied lysosomotropic agent that accumulates in the acidic organelles such as endosomes and lysosomes and neutralizes their pH 57. At present, it has been well studied that CQ has a wide-spectrum of anti-viral effects including anti-CoVs, anti-HIV, and anti-type A and B influenza viruses 58. The anti-viral effects of CQ and its analogs have been reviewed elsewhere 59. Here we would like to focus on the effect of CQ on CoVs, as summarized in **Table 2**. For instance, CQ has been shown to inhibit MERS-CoV replication *in vitr*o via a screening of an FDA-approved compound library 60. Treatment with CQ at a clinically relevant concentration, either before or after SARS-CoV infection into the Vero E6 cells, was found to be effective in suppressing viral infection, indicating its application in both prophylactic and therapeutic conditions 47. Similar results were also found in another lysosomotropic agent, ammonium chloride (NH_4_Cl) 47. It is known that the cleavage of the Spike Glycoprotein (S protein) by proteases is required for the SARS-CoV entry to the cells via a pH-dependent manner 45. Mechanistically, it is believed that the neutralization of endo-lysosomal pH by CQ inhibits the protease activities and prevents the cleavage of S protein and subsequently impairs the viral entry into the host cell. Interestingly, Wang *et al* showed that in cells treated with CQ, NH_4_Cl or Bafilomycin A1 (an endo/lysosomal V-ATPase inhibitor), the viral receptor ACE2 was trapped within perinuclear vacuoles 38, suggesting that these lysosomotropic agents may also affect the function of ACE2. Since ACE2 serves as the viral receptor for both SARS-CoV and SARS-CoV-2, such observations further support the notion for the potent anti-viral activity of those lysosomotropic agents. Indeed, a very recent study showed that CQ inhibits the SARS-CoV-2 infection at both entry and post-entry stages in Vero E6 cells 43.

In addition to its direct effects on CoVs, there is evidence of the combinational activity of CQ with other therapeutic agents for treatment of SARS, MERS and possibly COVID-19. For instance, He *et al* reported that CQ has synergistic effect on glucocorticoid signaling by stabilizing glucocorticoid receptor 61. Since glucocorticoid is one of the recommended therapy for severe SARS patients 62, it is possible that CQ may can be used for treatment of COVID-19 in combination of glucocorticoids and clinical trials are thus needed to test the efficacy of such combined therapy.

At present, CQ phosphate has been listed as a new therapeutic in the sixth version of “Guidelines for the Prevention, Diagnosis, and Treatment of COVID-19” issued by the National Health Commission of the People's Republic of China. And a number of clinical trials with CQ have been initiated in China 63. The current suggested dosage of CQ for COVID-19 is as high as 500 mg, with treatment not exceeding 10 days. However, the usage of CQ phosphate should be evaluated carefully as it may also have side effects such as retinopathy 64 and cautions should be taken for close monitoring of the potential side effect throughout the whole treatment period. The dosage should be reduced or stopped if reduction in haemoglobin concentration, lymphocyte count and platelet count, or the eyesight are observed. In addition, since CQ is the substrate of cytochrome P450 (CYP) enzymes which are responsible for the metabolism of multiple drugs, it might interfere with other medications such as digitoxin (a cardiac glycoside) and tamoxifen (used for treatment of breast cancer). More details of the toxicity and precautious of CQ can be referred elsewhere 65.

### Endosomal-lysosomal protease inhibitors

Cathepsins are endosomal and lysosomal cysteine proteases that play important roles in protein degradation in various cellular processes including both the endocytic pathway and autophagy 64. The role of cathepsins in viral infection was first identified by Huang *et al* and they found that one cysteine proteases inhibitor E64d and a specific cathepsin L inhibitor Z-FY(t-Bu)-DMK are able to block the SARS-CoV infection 48. K11777 is another cysteine protease inhibitor that has been reported to block the entry of SARS-CoV at the sub-nanomolar range 66. In addition, teicoplanin, a glycopeptide antibiotic and its derivatives inhibit the entry of MERS-CoV and SARS-CoV by inhibition of cathepsin L activity 55. Interestingly, a serine protease inhibitor camostat was known to inhibit transmembrane protease serine 2 (TMPRSS2) and effectively protected the mice against death caused by SARS-CoV infection 55. More importantly, a very recent study showed that camostat can also block the entry of SARS-CoV-2 by inhibiting ACE2 and TMPRSS2 67. Since camostat is already in clinical use for the treatment of chronic pancreatitis, suggesting its therapeutic potential for treatment of COVID-19.

### Inhibitors for clathrin-mediated endocytosis

As discussed earlier, clathrin-mediated endocytosis is one of the key mechanisms for viral entry into the host cells, including MHV 50, 52, 54, SARS-CoV 38, and MERS-CoV 53, 54. Therefore, chlorpromazine, an inhibitor for clathrin-dependent endocytosis, have been consistently found to possess significant inhibitory effect on the entry of those CoVs 39. In fact, chlorpromazine is a FDA-approved drug widely used for treatment of psychotic disorders such as schizophrenia 68. Importantly, it has been well established for its antivirus function for many types of viruses, including SARS-CoV and MERS-CoVs, as summarized in **Table 2**. At present, the clinical application of chlorpromazine in treatment of SARS and MERS has not been reported and it would be of interest to conduct clinical trials for testing the therapeutic effect of chlorpromazine on COVID-19.

In addition, two cardiotonic steroids ouabain and bufalin, selective inhibitors of the plasma membrane Na^+^/K^+^-ATPase, have been shown to inhibit the MERS-CoV infection at nanomolar concentrations via affecting the clathrin-mediated endocytosis pathway 54. Since both of them are also FDA-approved drugs and clinically available, it would be of interest to test them clinically for treatment of COVID-19.

## Summary and perspectives

The current ongoing epidemic of SARS-CoV-2 and COVID-19 worldwide has emerged as a significant global public health threat. While urgent regulatory measures in control of the rapid spread of this virus are essential, scientists around the world have quickly engaged in this battle by studying the molecular mechanisms and searching for effective therapeutic strategies against this deadly disease. At present, while the exact role of autophagy remains debatable, there is overwhelming evidence suggesting that the endocytic pathway plays a key role in mediating viral entry for many CoVs including SARS-CoVs, MERS-CoVs and possibly SARS-CoV-2. As a result, several inhibitors targeting the endocytic pathway appear to have the therapeutic potential in treatment of COVID-19, including a lysosomotropic agent CQ and a clathrin-mediated endocytosis inhibitor chlorpromazine 43, 63, 65, 68. Since both are FDA-approved and clinically available, clinical trials either as a single therapy or in combination with other anti-viral drugs are much needed.

## Figures and Tables

**Table 1:** Implication of autophagy in the infection of CoVs

Type of Coronavirus tested	Autophagy machinery tested	Infected Cells/Organs	Main findings	Refs
**Mousehepatitis virus (MHV)**	LC3 ATG12	DBT cells Mouse embryonic stem cell	Autophagy machinery are required for MHV replication	24
**SARS-CoV**	Endogenous LC3	Vero E6 cells	Viral replication-transcription complexes (RTCs) are co-localizing with endogenous LC3	25
**SARS-CoV**	Both Endogenous LC3 and GFP-LC3	Vero E6 cells	No evidence are observed for colocalization of LC3 or GFP-LC3 with the SARS-CoV RTCs	27
**Mousehepatitis virus (MHV)**	ATG5	Mouse embryonic fibroblasts (MEFs)	Deletion of ATG5 does not affect MHV replication	28
**Mouse hepatitis virus (MHV)**	ATG7	Mouse embryonic fibroblasts (MEFs)	Deletion of ATG7 does not affect MHV replication	29
**Bronchitis virus (IBV)**	ATG5 LC3	CHO cell line MEFs	Coronavirus replicase nsp6 protein induces autophagy	26
**SARS-CoV**	ATG5	Mouse embryonic fibroblasts (MEFs)	SARS-CoV replication is not affected in ATG5 KO MEFs	30
**Overexpression of membrane- associated papain-like protease PLP2 (PLP2-TM) of SARS-CoV and MERS-CoV**	LC3 Beclin1	HEK293T, HeLa and MCF-7 cells	Overexpression of PLP2-TM blocks autophagosomes-lysosomes fusion Beclin1 KD partially decreases coronavirus replication	31
**MERS-CoV**	Beclin1	Vero B4	MERS-CoV) blocks the fusion of autophagosomes and lysosomes Enhanced autophagy reduces the replication of MERS-CoV	32

**Table 2:** Involvement of the endocytic pathway and the respective inhibitors in CoV infection

Virus and cells tested	Part of endocytic pathway studied	Main findings	Effective inhibitors tested	Refs
MHV/ Mouse L cells, Sac2 cells, and DBT cells	Late endosome	MHV replication machinery co-localizes with late endosomal membranes	NA	44
SARS-CoV/Vero E6 cells	S protein-mediated entry	SARS-CoV entry requires acidification of endosomes	Balfilomycin A1, CQ, NH4Cl	45
SARS-CoV S glycoprotein/Vero cells	S-protein mediated entry	S-protein mediated entry is pH-dependent	Bafilomycin A1, NH4Cl	46
SARS-CoV /Vero E6 cells	Endo-lysosomal pH/cysteine protease	SARS-CoV entry requires acidification of endosomes	CQ, NH4Cl	47
SARS-CoV/Vero cells, 293T cells	Endo-lysosomal cysteine protease Cathepsin L	Cathepsin L is required for infection of cells with ACE2 expression	E64d, Z-FY-DMK	48
MHV/Murine fibroblast L2 and 17CL-1 cells	Endo-lysosomal cysteine protease Cathepsins	Endosomal proteolysis by cathepsins are required for viral entry	NH4Cl, CQ, Bafilomycin A1	49
MHV/ 17Cl-1 cells, LR-7 cells and DBT cells	Clathrin-dependent endocytosis	Infection by MHV is sensitive to lysosomotropic agents and inhibitors of endocytosis	Chlorpromazine, Bafilomycin A1, Concanamycin A, NH4Cl, Monensin	50
SARS-CoV/HepG2 cells	Clathrin-dependent endocytosis	Virus entry is mediated by clathrin-dependent endocytosis	Chlorpromazine, MβCD	39
SARS-CoV/Vero cells	Late endosome	Amiodarone inhibits late endosome to suppress SARS-CoV infection	Amiodarone	51
SARS-CoV/HEK293E cells	Clathrin- and caveolae-mediated endocytic pathway	Virus entry is mediated by a clathrin- and caveolae-independent endocytic pathway	NH4Cl, CQ, Bafilomycin A1	38
MHV/mouse astrocytoma DHT cells	Clathrin-or Caveolin-mediated endocytosis	MHV entry is via clathrin- but not Caveolin-dependent endocytosis	Chlorpromazine	52
MHV and MERS-CoV/ LR7 cells, HEK293T and Vero cells	Clathrin-mediated endocytosis	Entry of MHV is mediated by lysosomal proteases, while entry of MERS-CoV is mediated by furin	NH4Cl, CQ, Bafilomycin A1, Chlorpromazine, Monensin	53
MHV and MERS-CoV/ LR7 cells, HEK293T and Vero cells	Clathrin-mediated endocytosis	Cardiotonic steroids ouabain and bufalin inhibit infection of cells with MHV and MERS-CoV	Ouabain, Bufalin	54
MERS-CoV, SARS-CoV/HEK293T, A549, HeLa, etc	Late endosome-lysosome	Teicoplanin and derivatives inhibit Cathepsin L and block viral entry	Teicoplanin and derivatives	55
MERS-CoV, SARS-CoV/Vero81, Huh7, and Calu3 cells	Endosomal proteases	Cathepsin L-mediated S protein cleavage expands virus tropism	E64d, PCI (a proprotein convertase inhibitor, dec-RVKR-cmk)	56
SARS-CoV 2/ Vero E6	Endosomal pH	SARS-CoV-2 entry requires acidification of endosomes	CQ	43
